# Supplementary material for: From sequence to enzyme mechanism using multi-label machine learning
Source: BMC Bioinformatics. 2014 May 19;15:150. doi: 10.1186/1471-2105-15-150 (PMC4229970; doi:10.1186/1471-2105-15-150)
Supplement: Additional file 2 — Java code of ml2db. Additional file ml2db_code.tar.gz contains the Java source code to run the multi-label machine learning experiments and save the results to database. The code’s Javadoc is included. [file 1471-2105-15-150-S2.zip › additional file 2/ml2db/ml2db/doc/main/ml2db/al/utility/neighbours/LinearNNSearchSkipInstanceItself.html]

LinearNNSearchSkipInstanceItself


JavaScript is disabled on your browser.


- Overview
- Package
- Class
- Use
- Tree
- Deprecated
- Index
- Help

- Prev Class
- Next Class

- Frames
- No Frames

- All Classes

- Summary:
- Nested |
- Field |
- Constr |
- Method

- Detail:
- Field |
- Constr |
- Method


main.ml2db.al.utility.neighbours

## Class LinearNNSearchSkipInstanceItself

- java.lang.Object
- - weka.core.neighboursearch.NearestNeighbourSearch
  - - weka.core.neighboursearch.LinearNNSearch
    - - main.ml2db.al.utility.neighbours.LinearMultiLabelNNSearch
      - - main.ml2db.al.utility.neighbours.LinearNNSearchSkipInstanceItself

- All Implemented Interfaces:
  :   java.io.Serializable, weka.core.AdditionalMeasureProducer, weka.core.OptionHandler, weka.core.RevisionHandler

  ---

    

  ```
  public class LinearNNSearchSkipInstanceItself
  extends LinearMultiLabelNNSearch
  ```

  Class

  Version:
  :   25 Jul 2011

  Author:
  :   Luna De Ferrari ldf(at)st-andrews.ac.uk

  See Also:
  :   Serialized Form

- - ### Constructor Summary

    Constructors

    Constructor and Description

    `LinearNNSearchSkipInstanceItself(IdMultiLabelInstances instances)`
  - ### Method Summary

    Methods

    | Modifier and Type | Method and Description |
    | --- | --- |
    | `java.util.Vector<weka.core.Instance>` | `getNeighbours(weka.core.Instance instance, IdMultiLabelInstances instanceItselfAndOthers, int kNN, boolean skipItself)` |
    | `java.util.Vector<weka.core.Instance>` | `kNearestNeighbours(weka.core.Instance target, int kNN, boolean skipItSelf)` Returns k nearest instances in the current neighbourhood to the supplied instance. |

    - ### Methods inherited from class main.ml2db.al.utility.neighbours.LinearMultiLabelNNSearch

      `getLabelIndices, getNumberOfLabels, rebuild`
    - ### Methods inherited from class weka.core.neighboursearch.LinearNNSearch

      `addInstanceInfo, getDistances, getOptions, getRevision, getSkipIdentical, globalInfo, kNearestNeighbours, listOptions, nearestNeighbour, setInstances, setOptions, setSkipIdentical, skipIdenticalTipText, update`
    - ### Methods inherited from class weka.core.neighboursearch.NearestNeighbourSearch

      `combSort11, distanceFunctionTipText, enumerateMeasures, getDistanceFunction, getInstances, getMeasure, getMeasurePerformance, getPerformanceStats, measurePerformanceTipText, quickSort, setDistanceFunction, setMeasurePerformance`
    - ### Methods inherited from class java.lang.Object

      `equals, getClass, hashCode, notify, notifyAll, toString, wait, wait, wait`

- - ### Constructor Detail


    - #### LinearNNSearchSkipInstanceItself

      ```
      public LinearNNSearchSkipInstanceItself(IdMultiLabelInstances instances)
      ```
  - ### Method Detail


    - #### getNeighbours

      ```
      public java.util.Vector<weka.core.Instance> getNeighbours(weka.core.Instance instance,
                                                       IdMultiLabelInstances instanceItselfAndOthers,
                                                       int kNN,
                                                       boolean skipItself)
      ```


    - #### kNearestNeighbours

      ```
      public java.util.Vector<weka.core.Instance> kNearestNeighbours(weka.core.Instance target,
                                                            int kNN,
                                                            boolean skipItSelf)
                                                              throws java.lang.Exception
      ```

      Returns k nearest instances in the current neighbourhood to the supplied
      instance. It skips the instance itself.

      Parameters:
      :   `target` - The instance to find the k nearest neighbours for.
      :   `kNN` - The number of nearest neighbours to find.

      Returns:
      :   the k nearest neighbors

      Throws:
      :   `java.lang.Exception` - if the neighbours could not be found.


- Overview
- Package
- Class
- Use
- Tree
- Deprecated
- Index
- Help

- Prev Class
- Next Class

- Frames
- No Frames

- All Classes

- Summary:
- Nested |
- Field |
- Constr |
- Method

- Detail:
- Field |
- Constr |
- Method
